# Adsorption capability of brewed tea waste in waters containing toxic lead(II), cadmium (II), nickel (II), and zinc(II) heavy metal ions

**DOI:** 10.1038/s41598-020-74553-4

**Published:** 2020-10-16

**Authors:** Hakan Çelebi, Gülden Gök, Oğuzhan Gök

**Affiliations:** Environmental Engineering, Aksaray University, Aksaray, 68100 Turkey

**Keywords:** Environmental sciences, Environmental chemistry, Environmental impact

## Abstract

Recently, the search for low-cost eco-friendly adsorbents has become one of the main objectives of researchers. The aim of this study was to test the removal of four heavy metals, namely lead (Pb), zinc (Zn), nickel (Ni) and cadmium (Cd), from a simulated watery solution using brewed tea waste as a potentially suitable adsorbent. The effects of pH levels (2.0–6.0), adsorbent amount (0.1–5.0 g), contact times (1–150 min.) were examined throughout the adsorption process. The results of the experiments showed that the heavy metals elimination yields had an inverse relationship with pH and a linear relationship between the other parameters. The optimum pH for the removal of the heavy metals was between 4.0 and 5.0 in the case of the brewed tea waste. Equilibrium times of 2, 10, 30 and 5 min were required for the adsorption of Pb, Zn, Ni, Cd onto *Camellia sinensis*, respectively. Based on the results of this study it can be said that brewed tea waste has a high potential to remove heavy metals from aqueous solutions. The maximum adsorption capacities were calculated as 1.197, 1.457, 1.163 and 2.468 mg/g, for Pb, Zn, Ni and Cd, respectively, by fitting the equilibrium data to the Langmuir isotherm model.

## Introduction

The discharge of heavy metals and other toxic pollutants into waterways is one of the most significant and frequent detrimental effects of industrial activities that causes water pollution^1^. For this reason, many studies focusing on finding a solution to water contamination have been conducted in recent years^2–4^. Zhou, et al.^5^ evaluated data on 12 different heavy metal concentrations in surface waters (168 rivers and 71 lakes) between 1972 and 2017. In addition, they examined the total heavy metal concentrations of rivers and lakes in Asia, Europe and Africa. According to their results, the heavy metal concentrations in rivers in Africa and Asia were 45.04 and 17.75 µg/L for Cd, 83.82 and 92.70 µg/L for Pb, 1169 and 889.57 µg/L for Zn and, 131.69 and 54.84 µg/L for Ni, respectively. The heavy metal concentrations in European rivers, reached 5.69 µg/ L for Cd, 92.70 µg/L for Pb, 137.47 µg/L for Zn and 1338.99 µg/L for Ni. The assessment revealed that over the years’ heavy metal contamination had increased in aquatic environments and was at varying concentrations. Metal concentration in natural waters is closely linked with the geology of the region. Mineralized areas in particular have high metal concentrations due to their natural properties. In his study conducted in Turkey, Kacmaz^6^ stated that many streams, springs and deeper ground waters in mineralized regions contained high metal concentrations. In recent years, as a result of the development of important sectors such as agriculture, battery production, metal plating the discharge of heavy metals into the environment has increased both directly and indirectly. Hazardous heavy metals such as Cd, Pb, Zn and Ni in particular are used extensively in these sectors and cause significant problems in water and other receiving environments. The most harmful, toxic and carcinogenic heavy metals known are Pb, Cd, Zn and Ni. Table 1 shows the properties, lethal doses (LD_50_) and concentration values of common heavy metals identified^7–9^. Among the heavy metals polluting water environments the presence of hazardous heavy metals that are blacklisted by all countries of the world such as Pb, Cd, Zn and Ni are often detected^10^. These contaminants are generated frequently, thus, they accumulate in living organisms within the food chain and are liable for major health issues in both humans and animals^11^. It is paramount to check for heavy metals, which constitute one of the most hazardous and toxicant ingredients in wastewater^12–14^. Heavy metals are classed as metals and metalloids the atomic density of which exceed 4 ± 1 g/cm^3^. Up until now, many scientists have proposed a variety of potential physical and chemical processes for the removal of heavy metals from wastewaters, including ion exchange, chemical precipitation, electrochemical separation and reverse osmosis. The process of adsorption has often been preferred over other processes due to its low start-up costs, convenience, elasticity, high selectivity, ease of operation, environmental compatibility, insensibility to venomous components, and its major potential for the removal of hazardous contaminants. Many researchers have used natural materials such as carbon^15^, silica^16^, activated carbon, zeolite^17,18^ in the adsorption process. In some studies, eggshells^19^, olive mill waste^20^, peanuts, pistachio shells^21^, sugar beet bagasse^22^ and sunflowers^23^ have been used as the waste to remove heavy metals from wastewaters. The removal of heavy metal ions from waters using tea waste is essentially a three-step process: (i) adsorption, (ii) surface precipitation, and (iii) fixation^24^. Specific adsorption entails the surface complexation among functional groups and heavy metal ions in wastewater, and thus robust and permanent compounds are formed. C, H, N, O and S are the main elements of tea waste. This is why the functional groups on the tea waste surface area are predominantly made of surface hydroxyl groups, which bring surrounding ions together and supply protons to the liquid medium. When electron-pair acceptor metal ions are in question, surface complexes are formed between metal ions and hydroxyl groups^25^. Pourbaix diagrams were used to properly evaluate the metal diversity of Pb, Ni, Cd, Zn in the water samples subjected to chemical analysis. It was observed that for each metal analyzed, there was a large overlap between the chemical species predicted by the Pourbaix diagrams. The predominant species Pb, Ni, Zn, Cd in the aqueous phase depends on the pH and Pourbaix diagram. The main species present at pH ≤ 6 are Pb^2+^, Ni^2+^-Ni_2_H, Zn^2+^, Cd^2+^-CdOH^+^, while at pH ≥ 7 they are Pb(OH)_2_-PbO_2_, NiOH^+^, Zn(OH)_2_-ZnO_2_^2−^, and Cd(OH)_2_-CdO_2_^2–18,21–26^. The occurrence of these surface complexes, creating a three-dimensional phase during the removal of heavy metals from a medium by using tea wastes, can be described as surface precipitation. The diffusion of heavy metals into the tea waste is realized with the fixation mechanism, as the tea waste serves as an amorphous structure to which the method can be applied. The sorption kinetics are biphasic, with the first phase being the fast phase that is related with the larger area of available surfaces at the initiation of the process and the second phase being the slow stage phase which covered the diffusion in the micropores of the tea waste^24^.

**Table 1:** Concentration and specific properties of some heavy metals in waters.

Heavy metals	Permissible limits (mg/L)			Chemical properties				Toxicity mg/kg bw	
	WHO	USEPA	EU	VWR	PC	LogK_OW_	K_OC_	TWI	LD_50_
Ni^2+^	0.07	–	0.02	163	1.91	− 0.571	1.20	–	–
Zn^2+^	0.005	0.005	–	139	1.65	− 0.471	1.20	1.0 daily	20.2
Pb^2+^	0.001	0.0015	0.001	202	2.33	0.729	1.00	0.025	–
Cd^2+^	0.003	0.005	0.005	158	1.69	− 0.071	1.18	0.025 monthly	5.2

*VWR* Van der Walls Radius (10^–12^ m); *PC* Pauling Scale; *K*_*OC*_ Sorption coefficient; *Log*_*KOW*_ Octanol–water coefficient; bw: Bodyweight; *TWI* tolerable weekly intake for humans. *LD*_*50*_ Median lethal dose values obtained in mice or rats.

The adsorbent is a crucial parameter in the adsorption system. There are many parameters including pH and temperature of the medium, dose of the used adsorbent, and the allowed contact time that affect the yield of the adsorbents when removing various pollutants from wastewater. The diversity of low-cost adsorbents, such as industrial by-products, agricultural waste, food waste, types of activated carbons, clay minerals, biomass and polymeric materials, and zeolites, has been extensively investigated to be used for water and wastewater^20,27^. It is common for agricultural wastes to be used in batch adsorption experiments^28^. Brewed tea waste (BTW), which is a kind of agricultural waste, emerges in large quantities every year around the world. Interestingly, tea waste contains an insoluble cell wall consisting of cellulose, lignin, tannin and structural proteins with specific functional groups that are effective in forming physicochemical reactions with heavy metals and other contaminants and, thus, can be used to remove harmful substances from solutions and wastewaters^29^. In recent years, heavy metal and paint removal has been achieved with modifications of natural materials. As a result, the cost must be considered despite the high removal efficiency. Both waste control and the evaluation of wastes defined as waste in terms of treatment are of great importance in adsorption. Many studies have examined the adsorbent properties of waste tea leaves or tea factory wastes for the removal of hazardous substances from wastewaters^30^. However, there are limited studies that have been conducted on BTW. The present study focused on used tea residues that had not received any treatment in terms of activation, coating or modification. Furthermore, tea waste was selected as an adsorbent for heavy metal elimination and the possibility of using BTW in the elimination of Pb, Cd, Zn, Ni from watery environments was investigated. The adsorption behaviours of these metal ions in the tea waste were determined by observing the impacts of solution pH, BTW concentrations and contact time. Various other significant adsorption properties were also investigated. In addition, the main purpose of this study was to help scientists select suitable adsorbents for the removal of target heavy metals and facilitate the development of new adsorbents.

## Results

### BTW characterization

The composition of BTW was determined and the results are given in Table 2^31^. The surface structure of the BTW was not sufficiently developed. Therefore, as the BTW was used as an adsorbent, its adsorption capacity was negatively affected by the structure of its surface in addition to other mechanisms that played important roles in the rate of adsorption. In aqueous mediums, sample pH is one of the most important factors affecting zeta potential. Zeta potential readings taken at different pH values provide vital data about the sample’s surface and composition, such as the presence of functional groups. The zeta potential and the pH_ZPC_ (pH at point of zero charge) of the tea waste was − 20.58 mV and pH 2.45, respectively. The zeta potential of the BTW was always negative in the tested pH range of 2–10, indicating that the BTW was effective in attracting cations. These zeta potential values matched the data presented by Wan et al.^32^, who reported on the zeta potential of tea waste.

**Table 2:** Ultimate and proximate properties of BTW.

Proximate analysis (wt%, as received)		Ultimate analysis (wt%, dry basis)	
Moisture	7.20	Carbon	45.4
Volatile matter	70.29	Hydrogen	5.7
Ash	3.74	Nitrogen	2.9
Surface area	0.913 m^2^/g	Oxygen	46.0
Particle size	3.35–5.00 mm	K_2_O	8.23
Pore capacity	0.007 cm^3^/g	CaO	3.23

**Figure 1:**
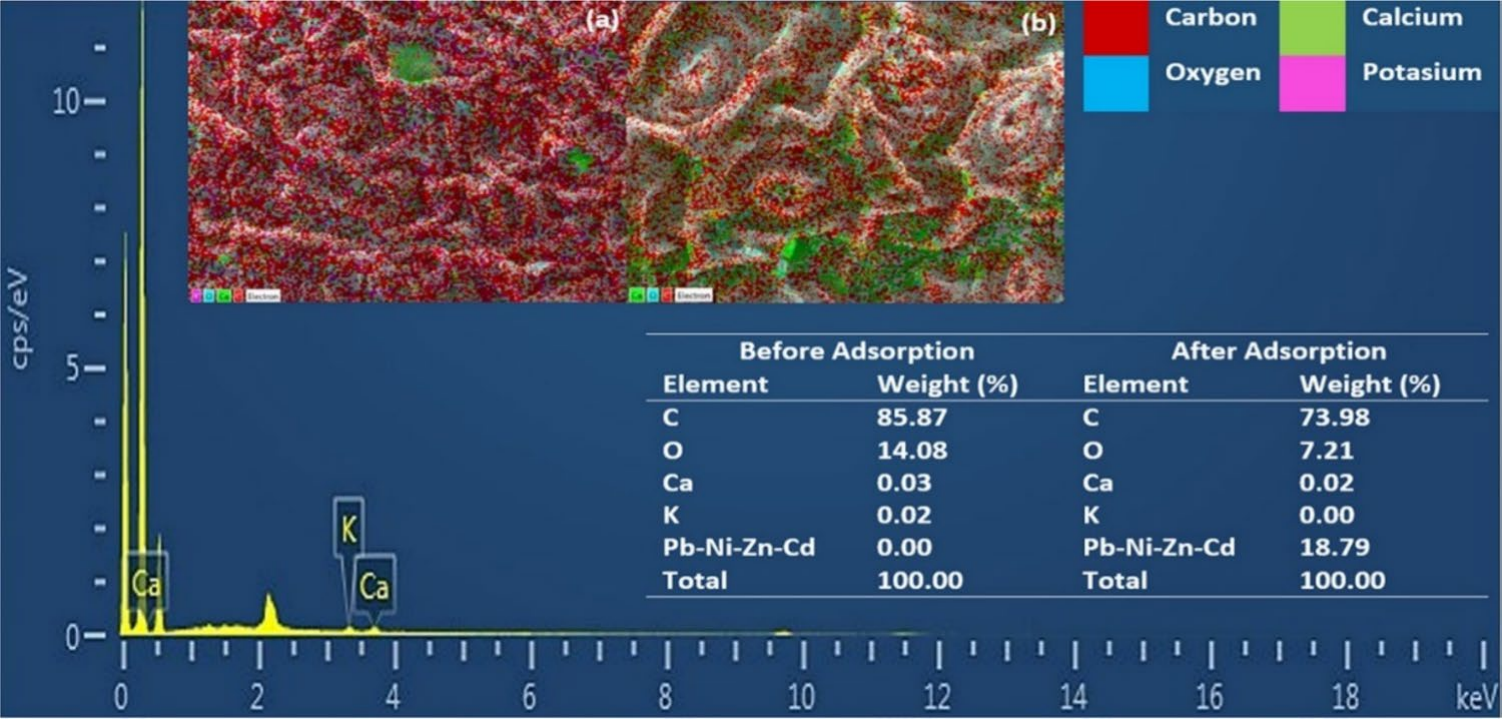
SEM and EDX (**a**) before and (**b**) after adsorption heavy metals by BTW.

**Figure 2:**
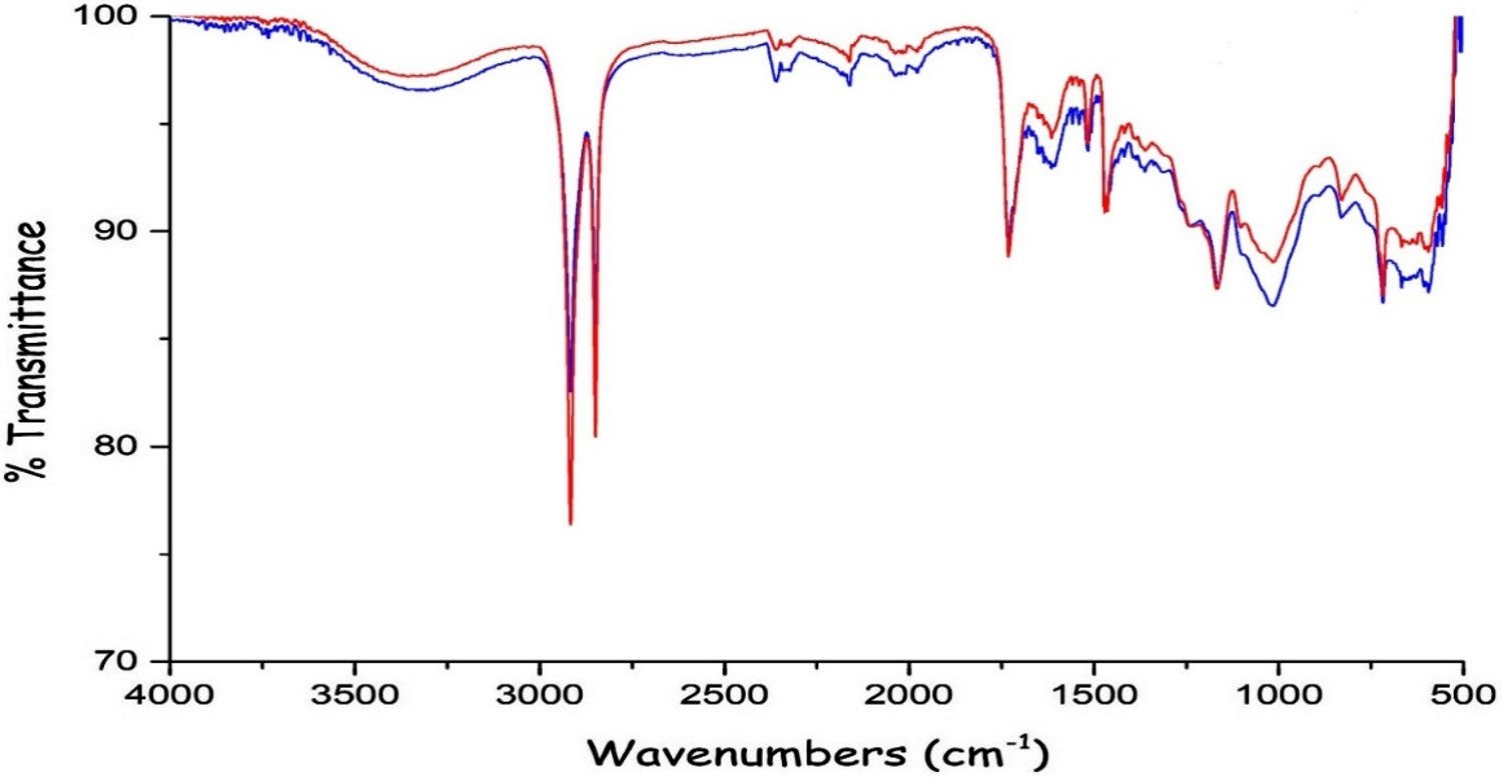
FTIR spectra before (red line), after (blue line) adsorption heavy metals by BTW.

A SEM micrograph of the BTW is shown in Fig. 1. As its main components are cellulose and hemicellulose, the BTW presented a stem structure. In addition, it showed a structure of heterogeneous and wide porous surface, which are favorable properties when using the substance in the adsorption of heavy metals. Generally, rougher surface area and broadly distributed pores can offer an effective surface area and more opportunities for the binding of heavy metal particles. Particle size determines the surface area of an adsorbent. Small particle size adsorbents create a larger surface area available for adsorption. According to the results, it can be attributed to both functional groups and particle size that adsorption may be limited to the outer surface. The main functional groups of the BTW are summarized in Table 3. The FTIR of the BTW and heavy metal clinging to the BTW are presented in Fig. 2. As a result of the FTIR analysis, it can be said that aliphatic C–H, C–O–H and C–O stretching functional groups in particular were involved in adsorption.

**Table 3:** Main functional groups of BTW and heavy metals loaded on BTW.

Before adsorption	After adsorption	Difference	Assignment
2916.62	2916.42	+ 20	Aliphatic C–H group
2849.35	2849.23	+ 12	Aliphatic C–H group
2160.69	2161.07	0	C=O stretching, C=O/C=C stretching
1615.38	1614.97	0	C–H alkanes in aromatıc rings
1516.44	1471.66	+ 44	–CH_3_ symmetric bending of CH_3_
1165.56	1167.43	− 2	C–O stretching
718.33	718.31	+ 2	C–O–H stretching

### Effect of pH

**Figure 3:**
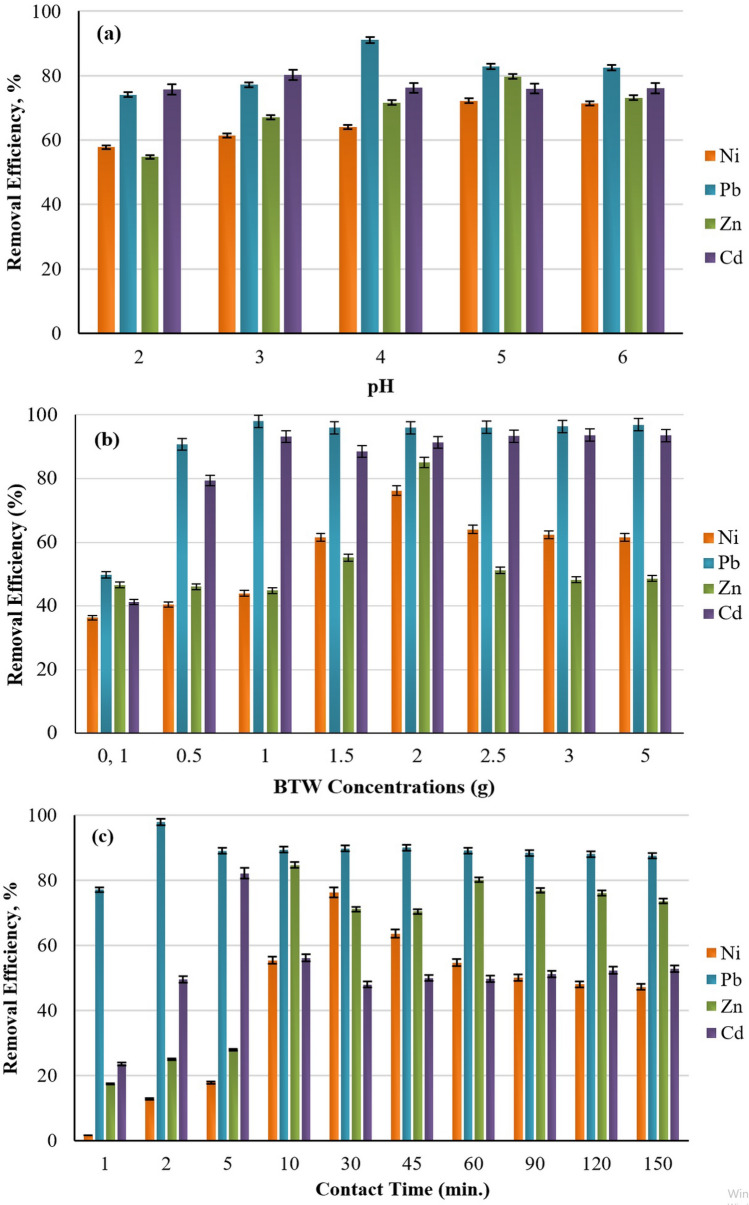
Impact of pH **(a)**, BTW concentration **(b)**, contact time **(c)** on the elimination of Pb, Cd, Ni, Zn ions.

In this study, the pH values were adjusted to be in the range of 2.0–6.0. As heavy metals cannot dissolve at neutral or basic pH values, determining the degree of metal contamination in water at pH ≥ 7 can be misleading. After pH 6, the formation precipitation of heavy metal ions as their own hydroxides may affect the adsorption results, therefore adsorption experiments were conducted in the pH range of 2–6. According to the Pourbaix diagram of Pb and Zn, the Pb^2+^ and Zn^2+^ ions precipitated as Pb(OH)_2_ and Zn(OH)_2_ at pH values greater than 6. However, at pH values lower than 6, the removal of the Pb^2+^ and Zn^2+^ ions by adsorption onto BTW was possible. The values pH > 6 are close to the values at which Cd(OH)_2_ precipitates. They also match the pH value for the Cd(OH)_2_ precipitation in a cadmium-water environment, as explained in the Pourbaix diagram. According to the Pourbaix diagram, Ni^2+^ was mainly present as uncharged Ni_2_H when the pH was lower than 6 and as NiOH^+^ and HNiO_2_^−^ when the pH (9–13) was higher^33,34^. As can be seen from Fig. 3a the removal percentage of the Pb and Cd ions was low when the pH was 2 or 3 as acidic conditions favour undissociated forms of functional groups. It was noted that when pH was 4, the removal percentage of the Pb and Cd ions increased significantly from 74.09 to 91.06% for Pb, 54.73% to 79.71% for Zn and 74% to 82% for Cd, respectively. The one-way ANOVA test showed that there was a significant relationship (p < 0.01) between Pb^2+^ removal percentages with pH changes. When the effects of pH value on the removal percentage of the metals were examined, it was found that the highest removal percentage for Ni and Zn was achieved at pH 5, and for Cd at pH 3 (p < 0.01). Changing the pH of the solution from 5 to 6 made no difference to the results. Wasewar et al.^35^ investigated a wide range of pH values from 2 to 12 and reported that the maximum Zn removal of 92% was obtained at pH 4.2. At lower pH values the H + ions compete with metal cations for the electrostatic surface charges in the system decreasing the percentage of sorption. These outcomes regarding the pH amount are also parallel to various studies that used acidic pH^14^. Basu et al.^1^ investigated the adsorption potential of sugar beet bagasse to remove Cr(VI) from aqueous solutions under optimum conditions including a pH of 5. They determined that removal efficiency was obtained to be 78.03%. Baby et al.^36^ observed that the adsorption of Pb, Zn, Cd on palm kernel shell is best at pH 2–6. The experimental results showed that the maximum removal percentages of Pb, Zn, Cd were 99.01%, 83.45%, and 84.23% respectively. The effect of pH on Ni adsorption was investigated at a pH between 2 and 6, a temperature of 20 °C and an initial Ni ion concentration of 104.3 mg/L. The level of metal adsorption was found to increase with the increase in pH from 2 to 6. The Ni ion showed maximum adsorption capacity at a pH of 5. At pH < 6 the predominant metal species were Ni^2+^ and Ni(OH)^+^. At values above pH 6, metal hydroxide Ni(OH)_2_ is likely to precipitate. At low pH, there were large amounts of H^+^ ions that effectively competed with the Ni ions for the adsorption sites and reduced the metal uptake capacity. Heavy metals are often present in their cationic state and are often more solvable and active in water mediums at neutral to low pH values. The pH of the medium can be a deciding factor on the adsorbent's surface charge^12^.

### Effect of BTW amount on the adsorption efficiency

As can be seen from Fig. 3b the increase in the BTW dose from 0.1 to 5 g in 100 mL, increased the removal efficiency of Pb, Ni, Zn and Cd by the BTW from 49.71% (1.488 mg/g) to 98.03% (1.197 mg/g), from 36% (1.652 mg/g) to 76% (1.457 mg/g), from 46.61% (2.532 mg/g) to 85.03% (2.468 mg/g) and from 41% (1.373 mg/g) to 94% (1.163 mg/g), respectively. These results were obtained at the optimal pH levels of 4 (Pb and Cd) and 5 (Ni and Zn) and the optimum contact times (Pb = 2 min, Ni = 30 min, Zn = 10 min and Cd = 5 min). This increase in yield was a result of the retention capacity of the active surface of the existing adsorbent for the Pb, Ni, Zn and Cd ions. In addition, the BTW amount can be decreased in accordance with the saturation of various regions on the BTW surface. From the results obtained, it can be observed that the significant level of less than 0.05 was obtained for BTW. Therefore, it can be concluded that the BTW was very effective in the removal of the Pb, Ni, Zn and Cd ions from aqueous solutions. The one-way ANOVA test showed a significant relationship (p < 0.01) between different BTW concentrations and removal percentages of the heavy metal ions. In addition, there was no statistically significant difference between the results obtained with 1.5 g and 2 g BTW doses in the percentage removal of the Pb ions (p > 0.01). Similar results were reported by Agarwal et al.^37^ for the adsorption of heavy metals using modified agricultural adsorbents. Thakur et al.^38^ investigated the elimination of Cu(II), Pb(II), Cd(II) and Zn(II) from wastewater by using activated rice husk with the batch adsorption process. In another study, walnut shells and the agricultural wastes of sunflowers, potatoes and canola have been used as biosorbents for the removal of heavy metals from aqueous solutions using batch experiments and the maximum adsorption capacity of the walnut shells was as follows: Cd (76.9 mg/g) > Zn (33.3 mg/g) > Ni (29.4 mg/g)^23^. Previous studies have concluded that Zn can be removed at an 80% removal efficiency by using black tea waste (20 g)^39^. The experimental studies conducted within the scope of the present study were in line with the results of relevant studies in the literature^40,41^.

### Effect of contact time and temperature

The adsorption of the Pb, Ni, Zn, Cd ions was gauged at certain contact times for the initial Pb, Ni, Zn, Cd concentrations of 96.71, 104.30, 98.70 and 108 mg/L, respectively. The removal efficiencies of the Pb, Ni, Zn, Cd ions increased up to 60 min and then remained almost constant (Fig. 3c). As can be seen from Fig. 3c the percentage of metal removal was initially higher (2 min—97.97%, 5 min—82%, 30 min—76%, 10 min—84.74%). This may be due to the structure of the initially present BTW for the adsorption of the Pb, Ni, Zn, Cd ions and a larger surface area. According to Fig. 3c, the metal uptake was fairly rapid for all concentrations examined and removal efficiency reached an equilibrium capacity of 50–97% of adsorption in 60 min. The one-way ANOVA indicated that there was a significant relationship (p < 0.01) between the removal percentages of the Pb, Ni, Zn, Cd ions and the increase in contact time. However, it was determined that similar percentage removal results were achieved for the Pb and Cd ions at certain contact times and that there was no statistical difference between the results (p > 0.01). Similar findings have been reported in the removal of heavy metal ions by tea wastes^42^. In addition, some studies in the literature have reported similar results for heavy metals using different agricultural wastes^43,44^. Yang et al.^29^ conducted Ni(II) removal experiments using modified green tea waste and determined the maximum removal efficiency of Ni(II) as approximately 75% in 10 min. Ghasemi et al.^45^ applied the adsorption method with tea waste for the removal of Cd from aqueous solutions. Their experimental results showed that the maximum removal of Cd with tea waste was 99.5% at 90 min. Temperature is also an important factor when assessing the behaviour of heavy metals and their eventual removal from solutions^46^. When evaluating the effect of temperature on the process of the removal of heavy metals, each adsorbent and metal ion must be tested specifically to define the effect of temperature changes on the overall adsorption. In the present study, the adsorption efficiency was investigated by increasing the temperature from 20 to 40 °C for 100 mg/L concentrations of Pb, Ni, Zn and Cd. Accordingly, as tea wastes exposed to high temperatures were used, there was no change in the heavy metal removal efficiencies in accordance with temperature changes (data not shown).

### Adsorption isotherm and kinetic models

**Figure 4:**
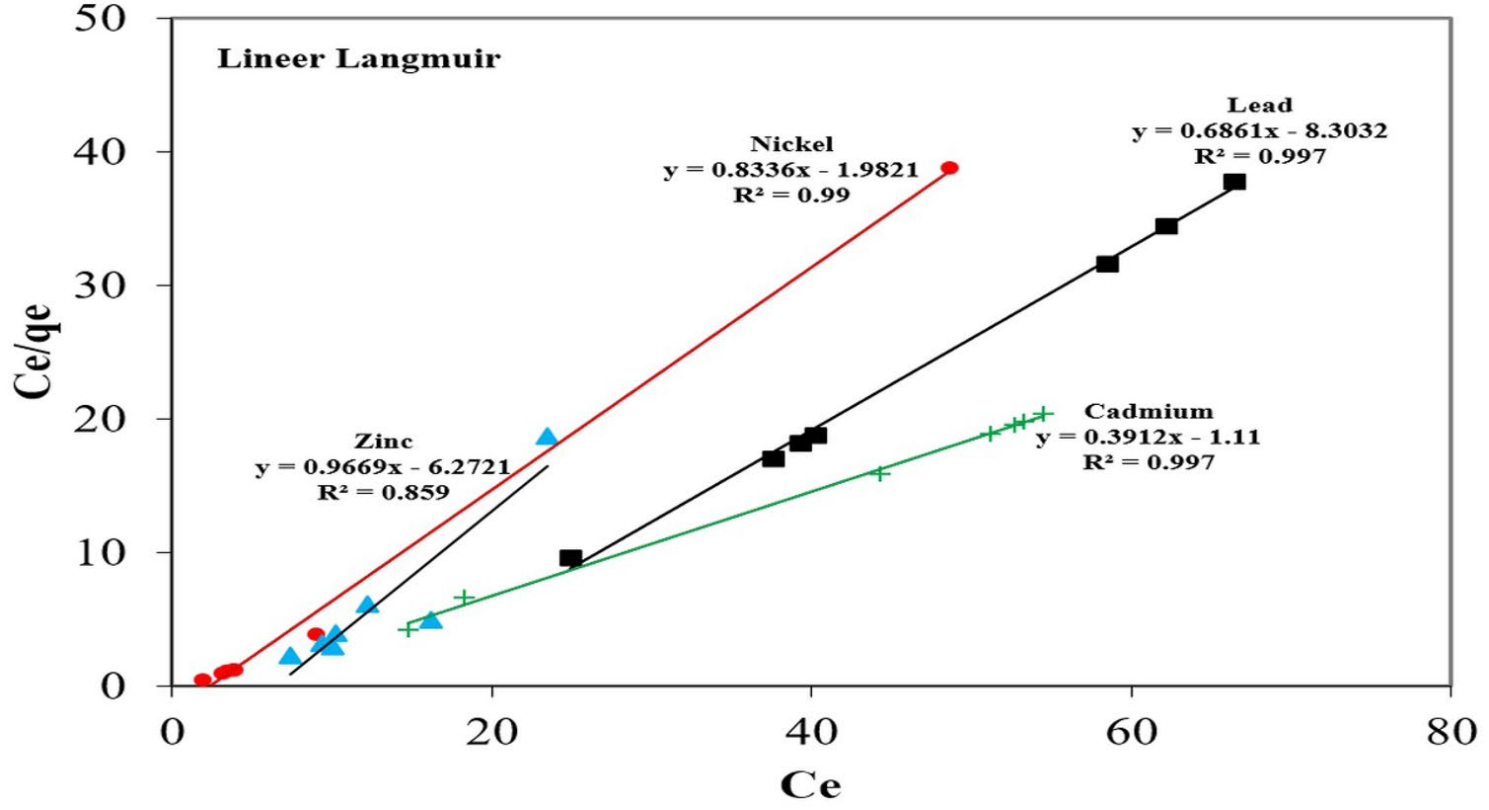
Langmuir isotherm curves of Pb, Cd, Ni and Zn adsorption by BTW.

**Figure 5:**
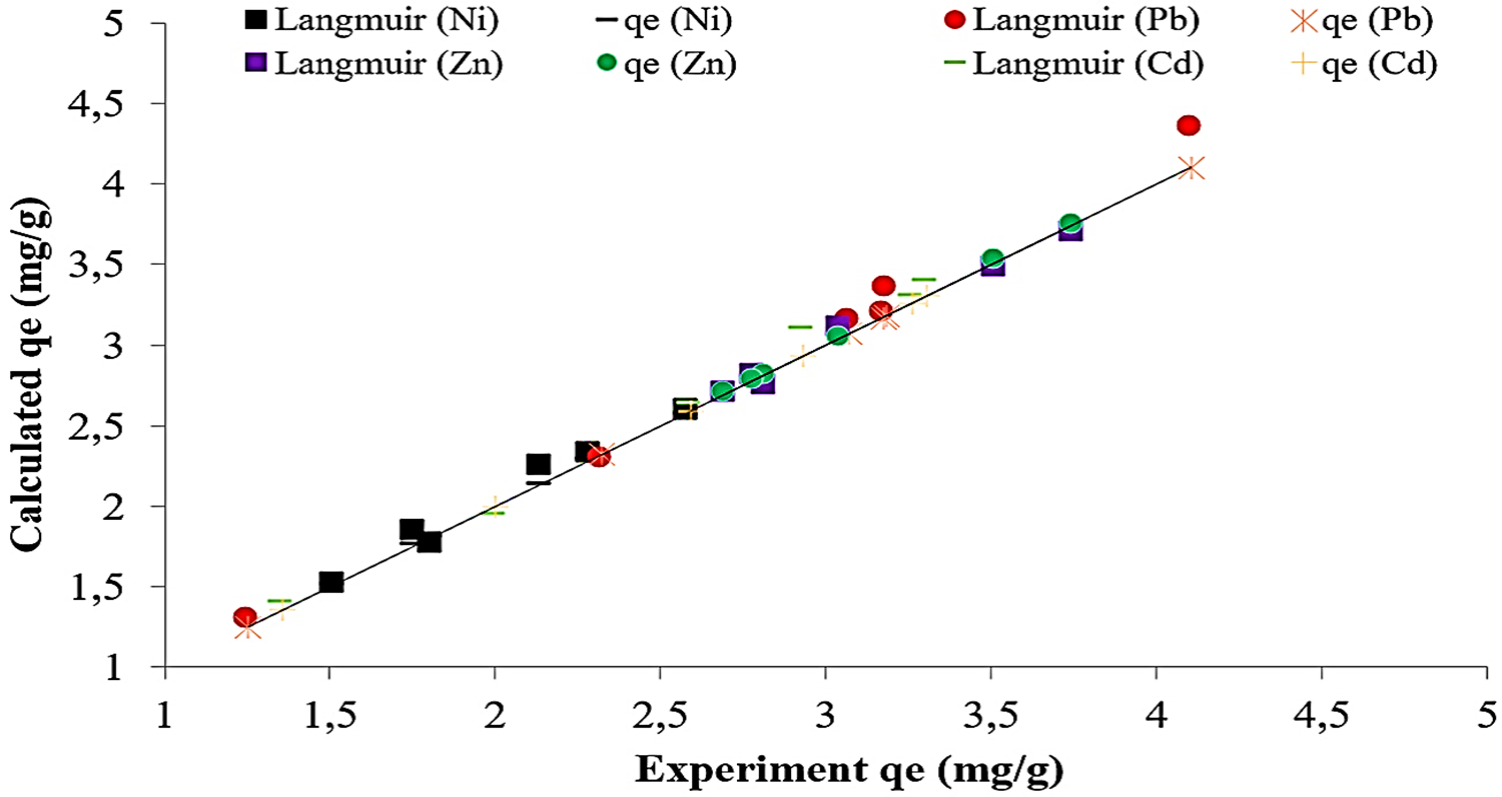
The plot between experimentally observed and calculated values of Langmuir.

In this study, the Langmuir, Freundlich, Temkin and Dubinin-Radukevich isotherms were employed to define the relationship between the adsorbate in the liquid phase and the adsorbate on the surface of the adsorbent. The regression correlation coefficient (R^2^) for the Temkin, Freundlich, Dubinin-Radukevich isotherms were relatively small in relation to that of the Langmuir isotherm. As can be observed from Table 3, the equilibrium adsorption data were better defined when the Langmuir isotherm model was used. The order of appropriateness of the isotherm models was as follows: Langmuir > Temkin > Freundlich > Dubinin-Radukevich (Fig. 4). As a result of adsorption studies, the Langmuir isotherm was found to be the most suitable for the heavy metals according to the R^2^ value. Therefore, only this isotherm model was used for the calculation of error functions (ErF)^47,48^. The results of the error functions are presented in Table 3. If the results from an isotherm are similar or close to four different function data, the error value should be small. As a result of the calculations, it was determined that the HYBRID error function gave the best result. The values of the Langmuir isotherm in this study indicated a positive interaction between the adsorbate and the BTW, which is valid proof of an ion-exchange mechanism taking place throughout the adsorption process (Table 4). Studies in the literature conducted on the adsorption of different heavy metals with various agricultural waste adsorbents have indicated that the removal of the heavy metal ions was fitted to the Langmuir isotherm^49,50^. 1/n in the Freundlich equation can be seen as a reflection of the difficulty of the adsorption behaviour. In general, when 1/n < 0.5 the experiment system is readily executed. The experiment system only becomes challenging when 1/n > 0.5^51^. In this study, 1/n was less than 0.5 when the Pb, Cd, Zn and Ni ions were adsorbed onto BTW, suggesting that the adsorption of the Pb, Cd, Ni and Zn ions with BTW was easily conducted. The Langmuir isotherm model was applied with ease to the adsorption process and the average maximum adsorption capacities of the Pb, Ni, Cd, and Zn ions were 1.197 mg/g, 1.457 mg/g, 1.163 mg/g and 2.468 mg/g, respectively. The results obtained were in accordance with the Langmuir model. This shows that the adsorption experiments were homogeneous. The scatter plots between the experimentally observed qexperimental (*q*_*e*, exp_) and model-calculated (*q*_*e*, cal_) values for the isotherms are presented in Fig. 5. The potential of the BTW was assessed by comparing the adsorption capacity of the Pb, Cd, Zn and Ni ions with various agricultural wastes adsorbents as shown in Table 5. It is clear that from the collected data that the BTW is effective in the removal of the Pb, Cd, Zn and Ni ions.

**Table 4:** Kinetics, isotherms constants and error functions for Pb, Cd, Ni and Zn.

Models	Parameters	Ni	Pb	Zn	Cd
Langm	q_m_ (mg/g)	1.457	1.197	2.468	1.163
	K_L_ (L/mg)	0.083	0.405	0.218	0.173
	R^2^	0.997	0.997	0.997	0.859
	R_L_	0.13	0.03	0.05	0.06
ErF	MPSD	16.827	20.951	12.696	14.498
	HYBRID	1.846	1.858	1.893	1.764
	NSD	15.192	15.214	12.928	10.367
	ARE	12.853	10.561	9.876	11.135
Frlich	K_F_ (mg/g)	2.538	5.189	6.175	4.981
	n	15.128	2.734	4.782	5.442
	R^2^	0.312	0.961	0.577	0.528
Temkin	A_T_	0.825	0.545	0.647	0.964
	R^2^	0.956	0.963	0.571	0.454
D-R	Ɛ (kj/mol)	1.036	7.808	2.148	9.569
	R^2^	0.926	0.571	0.983	0.389
PFO	k_1_ (1/min)	0.037	0.045	0.065	0.034
	R^2^	0.742	0.940	0.708	0.606
PSO	k_2_ (mg/g/min)	0.0006	0.032	0.026	0.017
	R^2^	0.921	0.983	0.967	0.933
ID	k_d_ (mg/g/min^1/2^)	0.937	2.507	0.806	2.933
	R^2^	0.385	0.474	0.643	0.514
Elovich	α (mg/g/min)	8.967	1.122	7.578	2.796
	β (g/mg)	0.49	0.91	0.47	0.90
	R^2^	0.716	0.917	0.619	0.765

**Table 5:** Adsorption capacities and yield of various tea waste based materials for the removal of Pb, Cd, Ni and Zn ions.

Adsorbent	metal	Optimum parameters					% Yield	Models	q_m_ (mg/g)	References
		m (g)	pH	C_0_ (mg/L)	t (min)	T (°C)				
MGTW	Ni^+2^	0.3	3.0	5	10	33	75	Langm/PSO	0.316	^29^
SCG	Zn^+2^	2.5	5.0	100	300	55	84.55	Frlich/PSO	5.25	^52^
WBT	Zn^+2^	20	5.0	100	250	25	80	Langm// Frlich /PSO	166.7	^39^
TW	Cd^+2^	10	5.0	5	90	20	99.5	Langm// Frlich /PSO	1.76	^45^
WS	Pb^+2^	1.0	4.0	100	2	20	90	Langm//PSO	9.912	^19^
TW	Cd^+2^	0.1	5.0	20	60	25	45	Langm// Frlich /PSO	16.87	^32^
TW	Pb^+2^	0.1	5.0	100	60	25	85	Langm// Frlich /PSO	33.49	^32^
BTW	Pb^+2^	1.0	4.0	100	2	20	97.97	Langm/PSO	1.197	In This Study
	Cd^+2^	1.0	4.0	100	5	20	84.74	Langm/PSO	1.163	
	Ni^+2^	2.0	5.0	100	30	20	82	Langm/PSO	1.457	
	Zn^+2^	2.0	5.0	100	10	20	76	Langm/PSO	2.468	

*MGTW* modified green tea waste; *TW* tea waste; *WBT* waste black tea; *SCG* spent coffee grounds; *OP* olive pomace; *WS* walnut shell.

**Figure 6:**
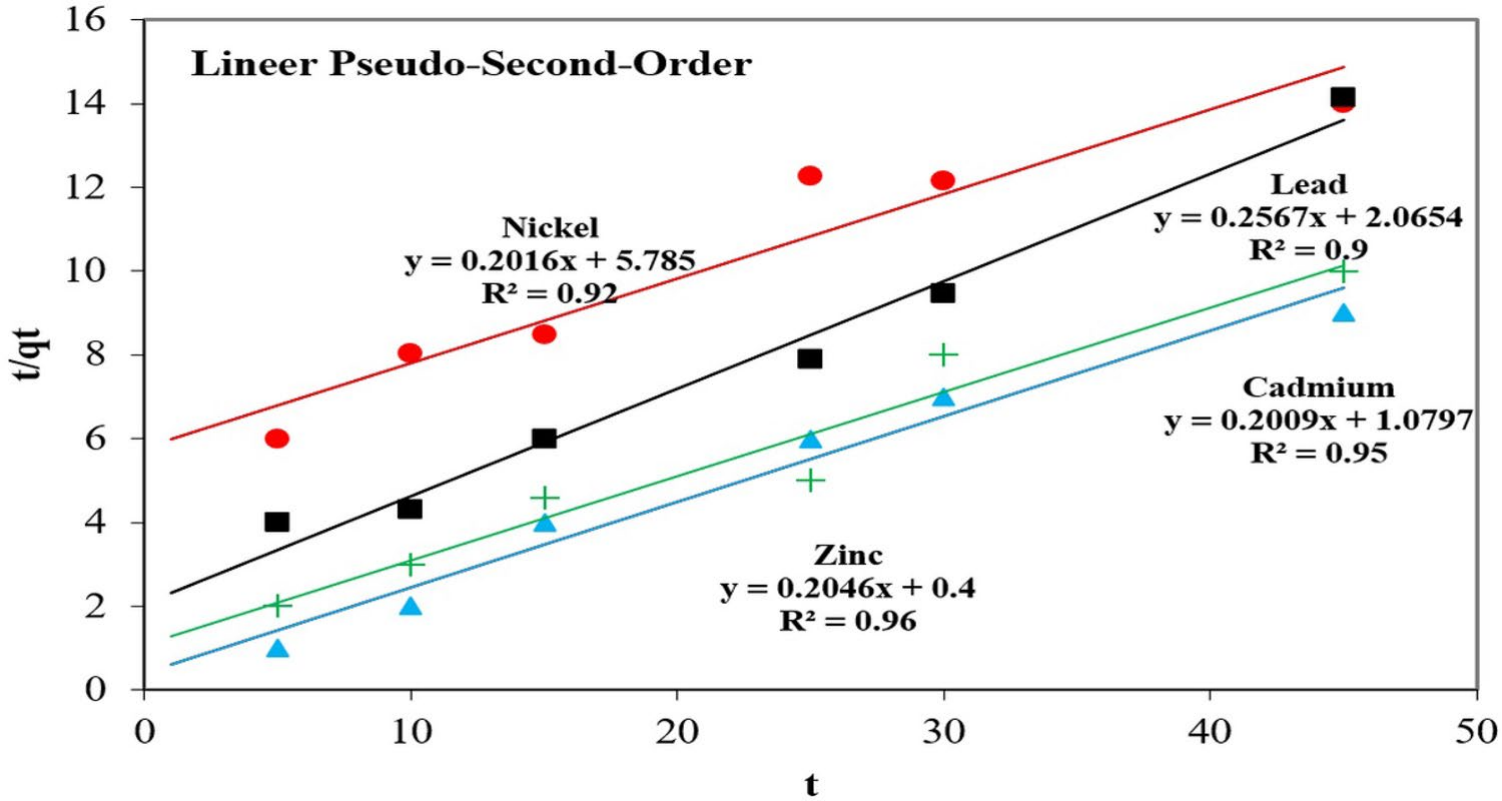
PSO kinetic curves of Pb, Cd, Ni and Zn adsorption by BTW.

The basic coefficients of the different kinetics are given in Table 4. Among the kinetic studies conducted, the Pseudo-second-order (PSO) (R^2^ = 0.99) was able to express the adsorption in this study clearly. The PSO model consisted of the external liquid film diffusion, surface adsorption and intra-particle diffusion processes^19,39,45,52^. Therefore, the results of this model can be accepted as a clearer and more reliable description of the adsorption mechanism between the BTW and the Pb, Cd, Zn and Ni ions. The PSO model further suggested that the process was one of chemisorption, which is the rate-limiting step for the adsorption of the selected heavy metals onto the BTW (Fig. 6). It means that the adsorption mechanism was controlled by electrostatic interaction in the solid/liquid medium with the reduced effect of the chemical reaction between the BTW and Pb, Ni, Cd, and Zn ions. The PSO model with higher R^2^ values better explains the fact that there may have been a speed limitation between the adsorption, the exchange of electrons or the BTW and the Pb, Ni, Cd, and Zn ions. According to the k_2_ constant, the adsorption ratio of the polluting cations onto the BTW occurred in the following sequence: Zn > Cd > Pb > Ni. According to previous studies that focused on the adsorption of heavy metals using various methods, the removal of the Pb, Cd, Zn and Ni ions is suitable for PSO kinetic modelling^19,39,45,52^. The findings of the present study regarding the Pb, Cd, Zn and Ni ions showed distinct correlation with the results of previous studies conducted on the topic.

## Conclusion

The BTW was determined as an efficient adsorbent in the removal of Pb, Ni, Cd, and Zn from wastewaters. The adsorption rate was rapid during the initial time periods. Equilibrium times of 2, 10, 30 and 5 min were required for the adsorption of Pb, Zn, Ni, Cd ions onto BTW, respectively. The adsorption process explained by the Langmuir isotherm had a monolayer adsorption capacity of 1.197 mg/g, 1.457 mg/g, 1.163 mg/g and 2.468 mg/g, for the Pb, Ni, Cd, and Zn ions, respectively. HYBRID was found to be suitable for Langmuir in the adsorption of the heavy metals onto BTW. In addition, as a result of the kinetic studies it was determined that the PSO model was the most suitable kinetic model for the Pb, Ni, Cd, and Zn ions. The maximum adsorption of Pb and Cd was achieved at pH 4, while for Ni and Zn it was achieved at pH 5. The highest removal rates were in the order of Pb (97.97%) > Ni (82%) > Zn (76%) > Cd (84.74%). The adsorption rate and capacity were dependent on the BTW dose, solution concentration and particle size. The results of this study showed that BTW was a more efficient adsorbent compared to some of the treated biomass materials and that BTW could be suitably used as an alternative and effective adsorbent material for the removal of Pb, Ni, Cd, and Zn ions from synthetic wastewaters.

## Methods

### Adsorbent preparation and characterization

Before being used as an adsorbent the BTW was required to go through several preliminary steps, such as grinding, sieving and washing. The BTW was obtained from food units on the campus of Aksaray University. The collected BTW were washed and then boiled at 100 °C for colour removal. Then, they were then dried in sunlight for an average of two days. The naturally dried BTW were stored in a 60 °C incubator for 48 h. Next, the BTW was pulverized by a mixer and sieved. Spectra in the range of 600–4000 cm^−1^ were determined by the Fourier transform infrared spectrometer (FTIR) (Thermo Scientific-Nicolet iS5) to identify the functional groups of the BTW samples. Scanning electron microscope (SEM) and energy-dispersive X-ray spectroscopy (EDS) analysis (Hitachi-SU 1510) were performed using a microscope equipped with an energy dispersive analytical system operating at an acceleration voltage of 5–15 kV and an EDAX Apollo detector. The surface area measurement was performed using a Quantachrome-Quadrasorb Evo 4 brand device with the Brunauer, Emmett, and Teller (BET) method in a 77 K liquid nitrogen medium based on the N_2_ gas technique. The elemental composition for the adsorbent material was determined using an elemental analyzer (Perkin-Elmer 2400). Proximate (ash, moisture, etc.) experiments were performed according to the ASTM D 2866-94^53^ and ASTM D 2867-95^54^ methods. A zeta meter was used to measure the zeta potential of the BTW (Pen Kern Laser Zee 3.0, USA). For this purpose, 0.05 g of the BTW was diluted in 500 mL of deionize water with 0.01 mol/L NaNO_3_ solution.

### Batch adsorption experiments

In this study, the performance of the adsorption process using the BTW for the removal of heavy metals from aqueous solution was investigated on a laboratory scale with a batch test system. 100.0 mg of the BTW was placed in 250 mL Erlenmeyer flasks that contained 100 mL solution with a concentration of the metals. All of the metal ions were studied individually. The initial pH value of the prepared synthetic wastewater was between 5.8 and 6.1. The pH value of the solution was changed using 1.0 M HNO3 or NaOH solution as needed during the experiments. The pH values were adjusted to be in the range of 2.0–6.0. Subsequently, the flasks were shaken in a shaking incubator (Model G-25) with a thermostat at 150 rpm for 24 h at 20 °C. Afterwards, the samples were filtered to extract the fine BTW particles and analyzed to detect the metal ions present in the fluids. The overall uptake of the studied metals was determined by measuring the metal mass in the fluid before and after the tests. Every batch was performed twice to increase the reliability of the data. The optimum pH and time was determined at the constant values of initial Pb, Ni, Zn and Cd concentrations and adsorbent dosages. Finally, the isotherms and kinetics of the adsorption of the heavy metals onto the BTW were determined separately. All of the chemicals used were purchased from Merck and Sigma. The contaminated solutions were prepared by dissolving the heavy metal salts in deionized water. The Pb, Cd, Zn and Ni ions were used as the model pollutants. Four different stock solutions were prepared for the selected heavy metals. Adsorption studies were performed separately for each ion. The 1000 mg/L stock solutions containing Cd, Pb, Ni and Zn were obtained from 3CdSO_4_·8H_2_O, Pb(NO_3_)_2_, NiCl_2_.6H_2_O and Zn(NO_3_)_2_ (Sigma-Aldrich Chemie GmbH, Germany), respectively. The working solutions of 100 mg/L Pb, Ni, Cd and Zn were prepared from the stock solution by serial dilutions. Although the concentrations of heavy metals in natural waters are generally below 100 mg/L, this study demonstrated that the existing adsorption process can also be used in industrial wastewater with high heavy metal concentrations such as the battery and metal coating industries. In addition, the Pb, Ni, Cd and Zn ion concentrations of the synthetically prepared wastewater were used at 100 mg/L concentration to represent real industrial wastewaters. The concentrations of the heavy metals in the studied samples were measured using a standard method with an inductively coupled plasma‐optical emission spectrometer (ICP-OES) (Shimadzu-1700, Japan). All laboratory studies were repeated three times and the average numbers were used in the calculations. The removal and adsorption rates were calculated using the following equations:

1 $${\mathrm{q}}_{\mathrm{e}}=\frac{\left({\mathrm{C}}_{0}-{\mathrm{C}}_{\mathrm{e}}\right)\mathrm{ x V}}{1000\mathrm{ x m}}$$

2 $$\mathrm{Y }(\mathrm{\%})=\frac{\left({\mathrm{C}}_{0}-{\mathrm{C}}_{\mathrm{e}}\right)}{{\mathrm{C}}_{0}}\mathrm{x}100$$

In these equations, Y is the rate of removal of Pb, Cd, Ni and Zn ions (%). C_0_ and C_e_ are respectively the initial and final concentrations of Pb, Cd, Ni and Zn (mg/L). m represents the mass of BTW (mg). The volume of the solution (mL) is indicated with V, where qe is the amount of adsorbed Pb, Cd, Ni and Zn ions by the BTW (mg/g).

### Assessment of different models and error functions for selected heavy metals

The Freundlich, Langmuir, Dubinin-Radushkevich and Temkin isotherms were used extensively to define the relationship between the Pb, Cd, Ni and Zn ions in the synthetic water with BTW. The isotherm species of Freundlich (F) (3), Langmuir (L) (4), Dubinin-Radushkevich (D-R) (5), Temkin (T) (6), separation factor (R_L_) (7) were placed to the empirical results^55–59^.

3 $${q}_{e}={K}_{F}\sqrt[n]{{C}_{e}}$$

4 $${q}_{e}=\frac{{q}_{m}{K}_{L}{C}_{e}}{1+{K}_{L}{C}_{e}}$$

5 $$In\,\,{q}_{e}=\mathrm{In}\,\,{q}_{m}-\beta {\epsilon }^{2}$$

6 $${q}_{e}=\frac{RT}{{b}_{T}}\,\,\mathrm{ln}\left({A}_{T}{C}_{e}\right)$$

7 $${\mathrm{R}}_{\mathrm{L}}=\frac{1}{1+{\mathrm{K}}_{\mathrm{L}}{\mathrm{C}}_{0}}$$

8 $${q}_{t}={\text{q}_{e}} \left(1-e^{{{-k}_1}t} \right)$$

9 $${q}_{t}=\frac{{K_{2}}{q_{e}^{2}}t}{1+{\text{k}_{2}}{q_{e}}t}$$

10 $${\text{q}}_{\text{t}}=\frac{1}{\beta}\,\,\mathrm{ln}\left(\alpha \beta \right) + \frac{1}{\beta}\,\,\mathrm{ln}\left(t\right)$$

11 $${\text{q}}_{\text{t}}= {\text{K}}_{\text {d}} \text{t}^{0.5}$$

C_e_: balance amount of Pb, Cd, Ni and Zn ions (mg/L). q_e_: the number of heavy metals-BTW at balance (mg/g). qm: the maximum adsorption capacity (mg/g), K_L_: Langmuir constant (L/mg). K_F_ and 1/n: Freundlich constants. Ɛ and R (8.314 J/mol K) specific constants and T is a temperature (K). Temkin isotherm equilibrium binding constant is A_T_ (L/mg), b_T_ (J/mol): Temkin isotherm constants. Adsorption kinetics are used to examine the changes in adsorption in a time period and can provide information about the experiment system. In our research, various non-linear kinetics suitable for the system were tested^60^. Pseudo-first order (PFO) (Eq. (8)), pseudo second-order (PSO) (Eq. (9)) Elovich model (Eq. (10)) and intraparticle diffusion (ID) (Eq. (11)): q_e_, q_t_: the amounts of heavy metals adsorbed (mg/g) at balance. k_1_ (1/min): the PFO parameter. k_2_, k_d_: the PSO parameter. a (mg/g/min): The chief adsorption rate.

### Statistical analysis

SPSS 22.0.0 software (SPSS Inc., Chicago, USA) was used to gain insight into the effectiveness of the BTW in removing the heavy metal ions from the aqueous solutions. For this purpose, the one-way analysis of variance (ANOVA) test was applied at a 99% confidence interval (p < 0.01). Before the ANOVA test, all data passed the normality test. Then, the Duncan test was carried out to compare the means of each parameter. A minimum of three repeated measurements were performed to detect variability. The results obtained are presented in the bar charts as mean and standard deviation (Mean ± sd).

## Supplementary information

Supplementary Information.
