# Supplementary material for: Adsorption capability of brewed tea waste in waters containing toxic lead(II), cadmium (II), nickel (II), and zinc(II) heavy metal ions
Source: Sci Rep. 2020 Oct 16;10:17570. doi: 10.1038/s41598-020-74553-4 (PMC7567786; doi:10.1038/s41598-020-74553-4)
Supplement: Supplementary file 1 — Supplementary Information. [file 41598_2020_74553_MOESM1_ESM.pdf]

Adsorption capability of brewed tea waste in waters containing toxic lead(II), cadmium (II), nickel (II), and zinc(II) heavy metal ions

Hakan Çelebi*, Gülden Gök, Oğuzhan Gök

Aksaray University, Environmental Engineering, Aksaray, 68100, Turkey

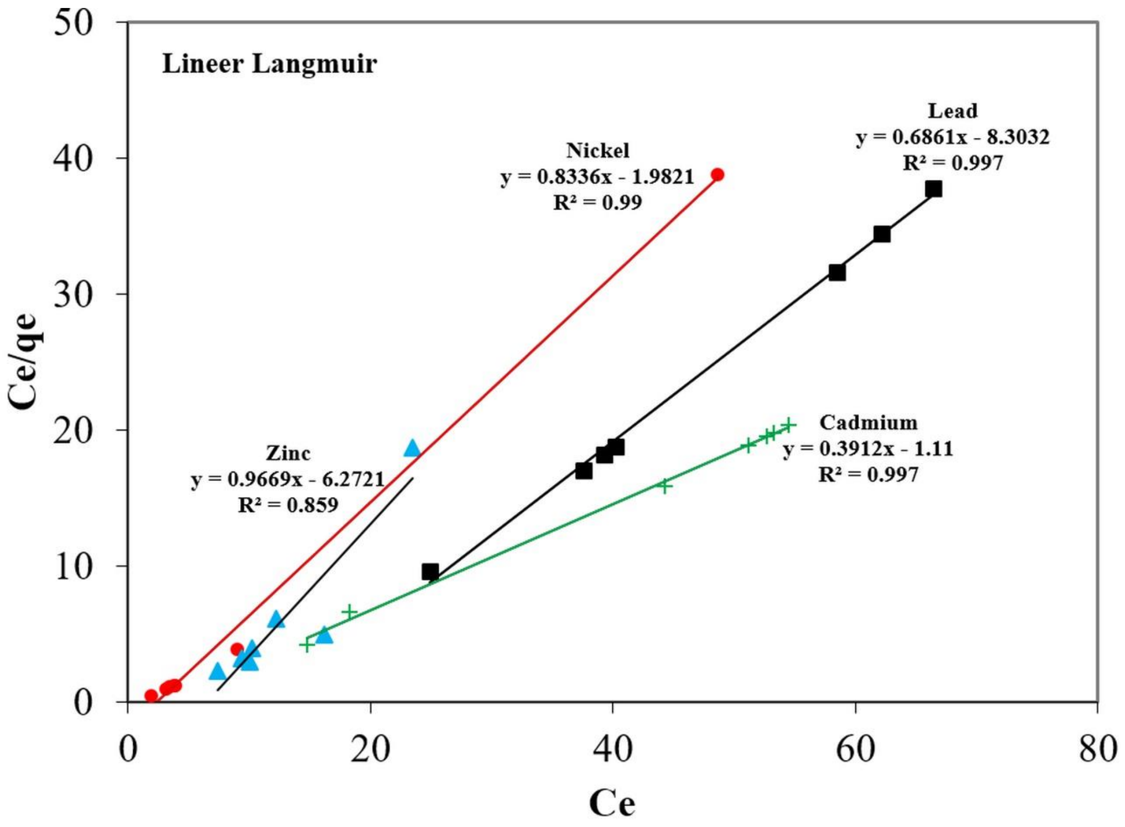

Sup. Figure 1. Langmuir isotherm curves of Pb, Cd, Ni and Zn adsorption by BTW

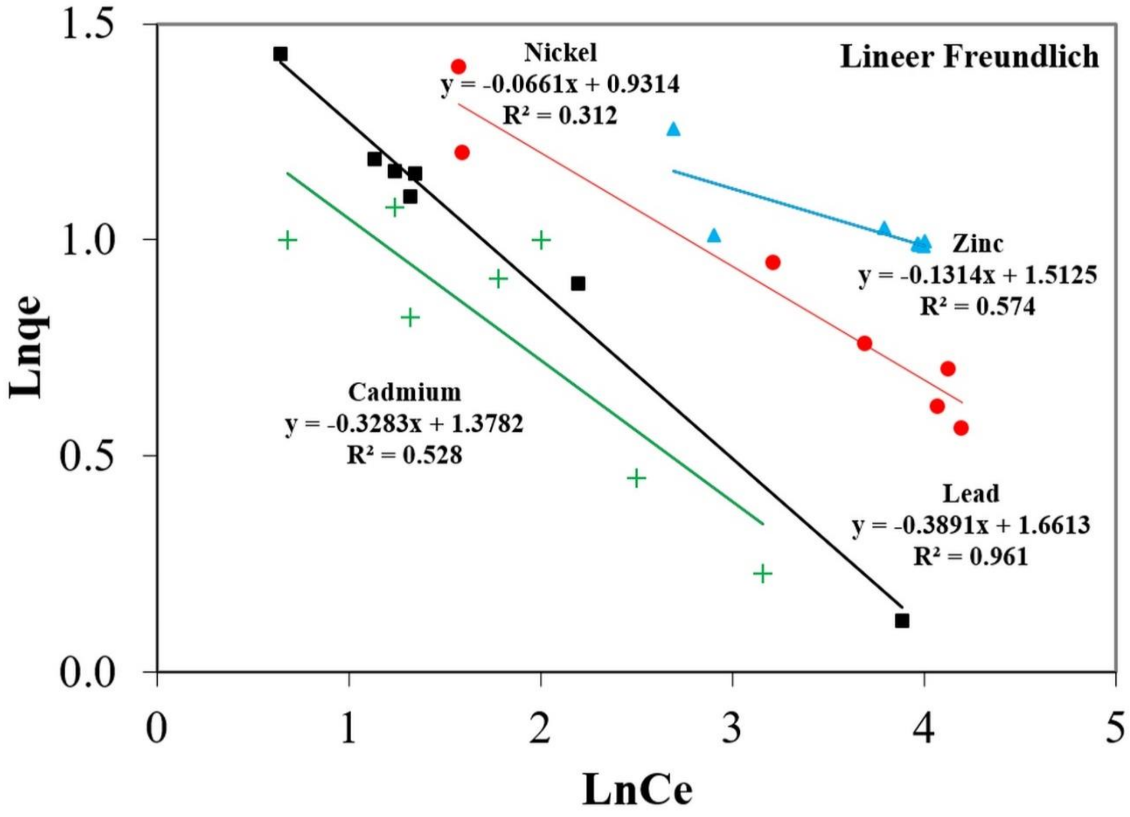

Sup. Figure 2. Freundlich isotherm curves of Pb, Cd, Ni and Zn adsorption by BTW

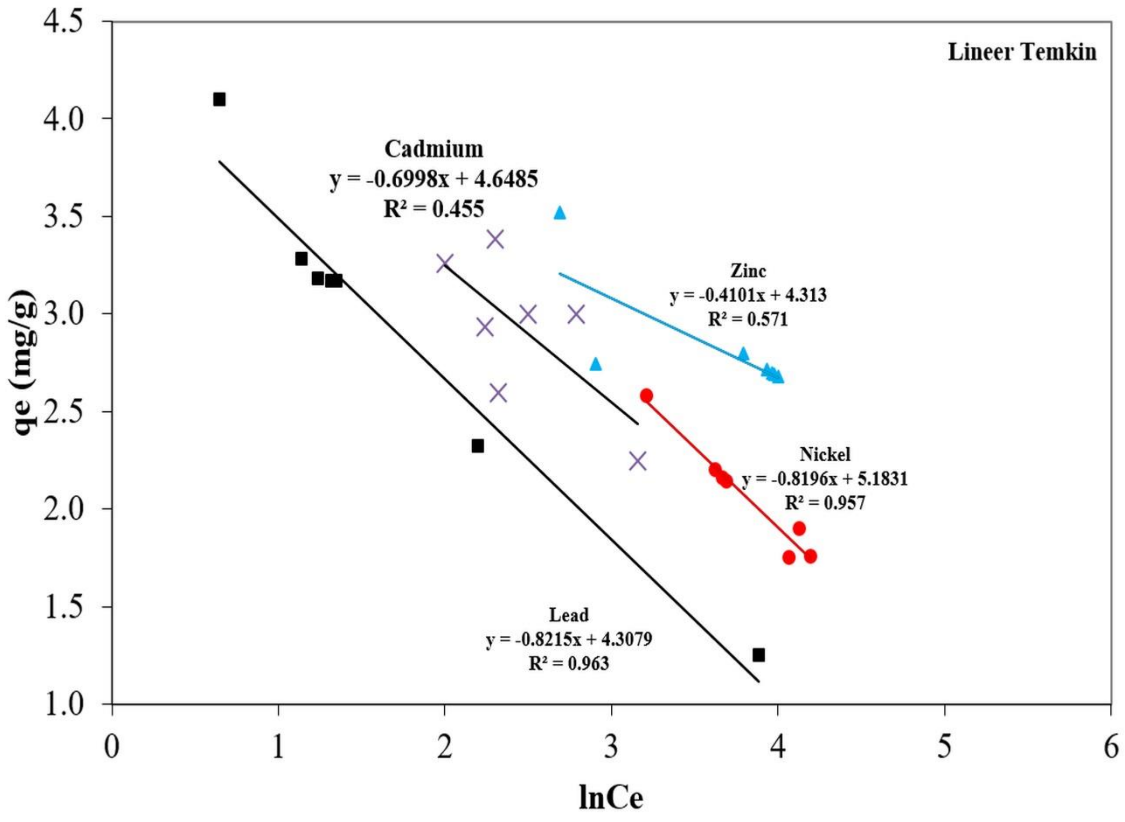

Sup. Figure 3. Temkin isotherm curves of Pb, Cd, Ni and Zn adsorption by BTW

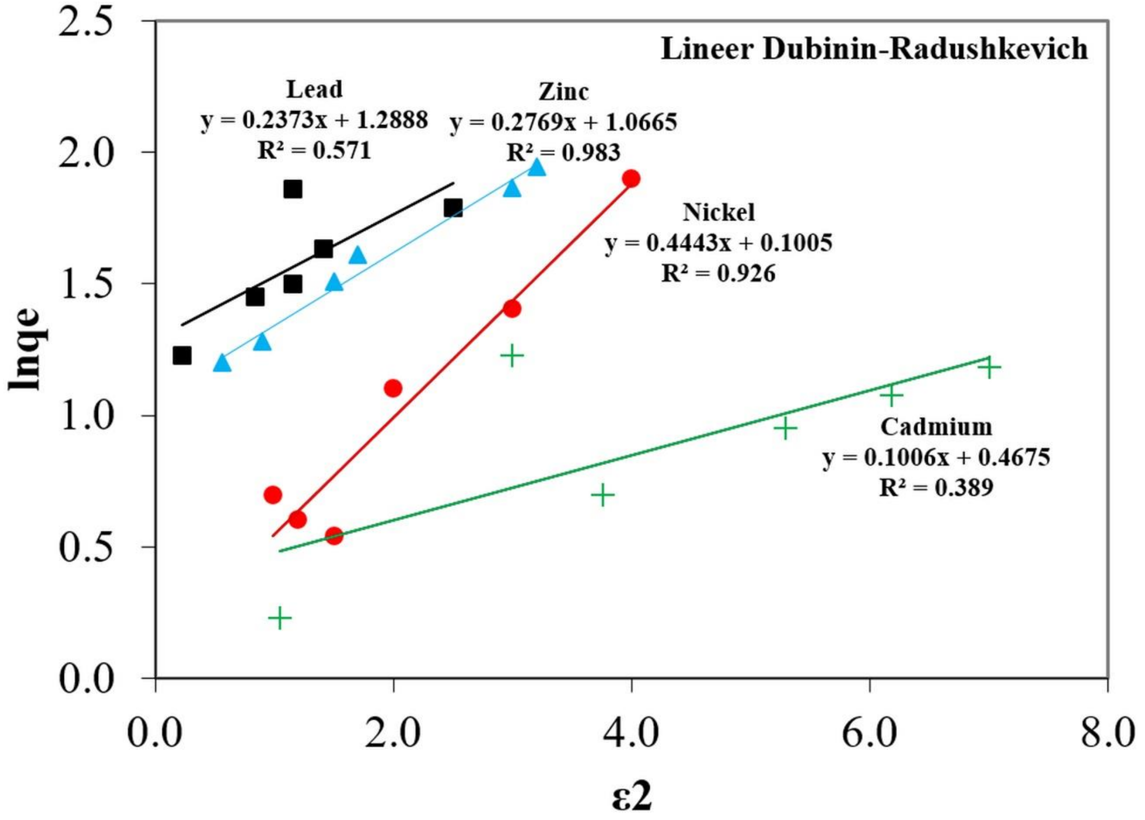

Sup. Figure 4. Dubinin-Radushkevich isotherm curves of Pb, Cd, Ni and Zn adsorption by BTW

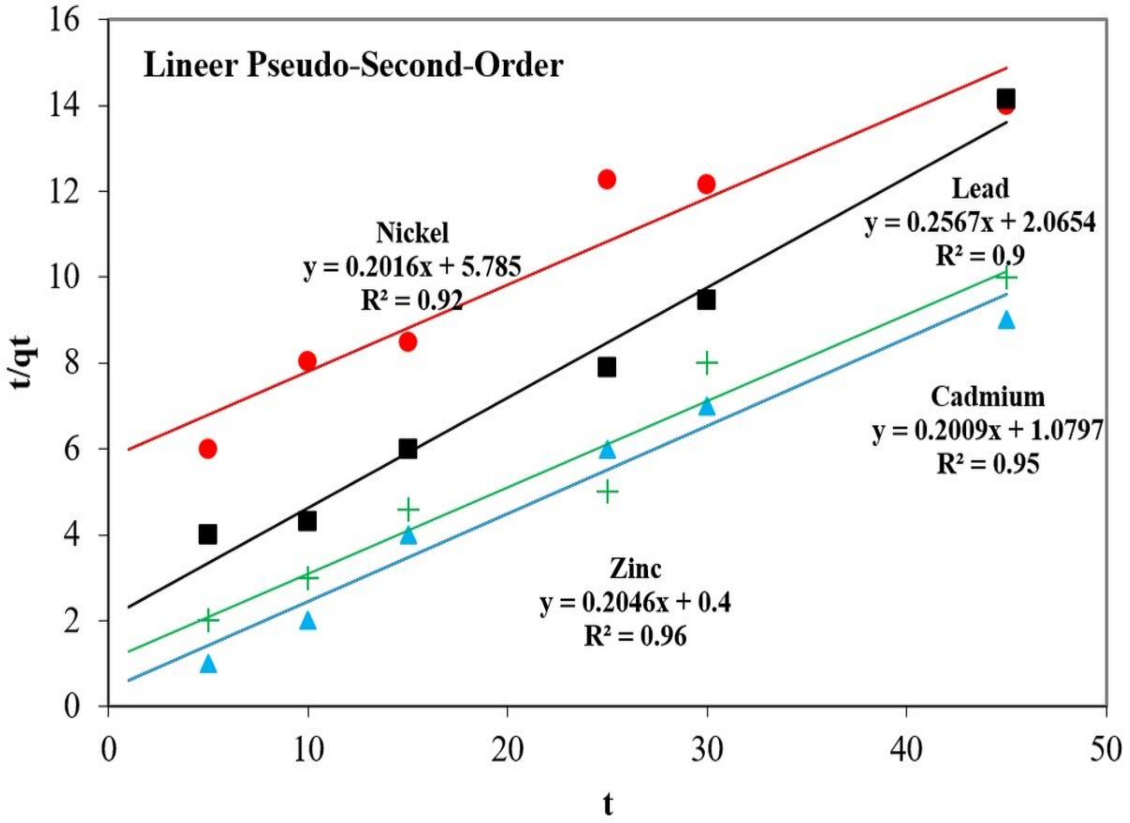

Sup. Figure 5. PSO kinetic curves of Pb, Cd, Ni and Zn adsorption by BTW

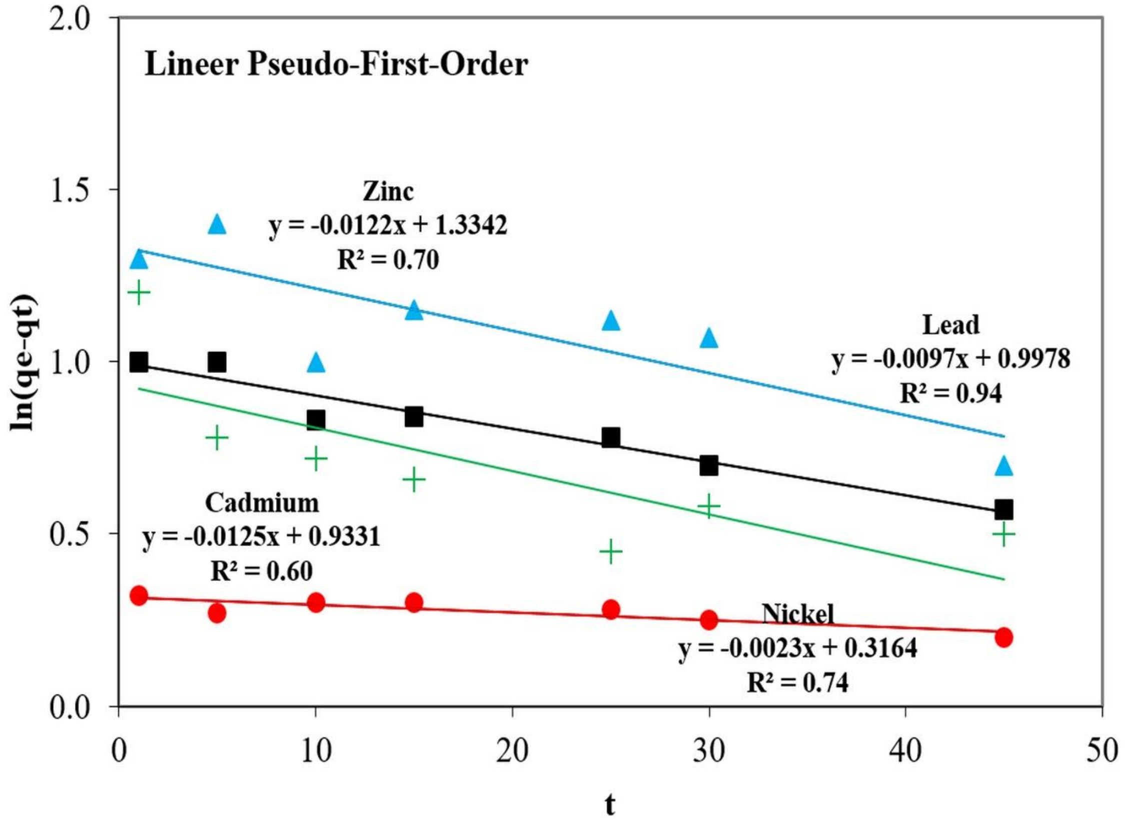

Sup. Figure 6. PFO kinetic curves of Pb, Cd, Ni and Zn adsorption by BTW

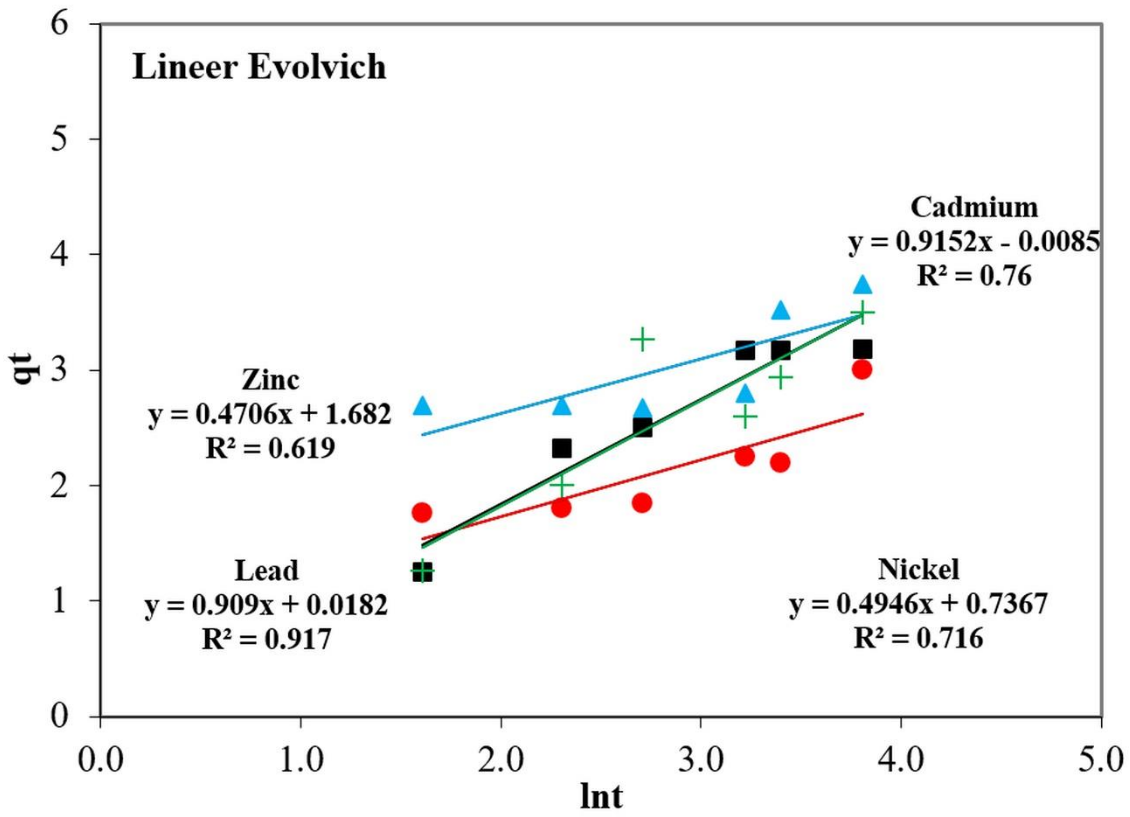

Sup. Figure 7. Evolvich kinetic curves of Pb, Cd, Ni and Zn adsorption by BTW

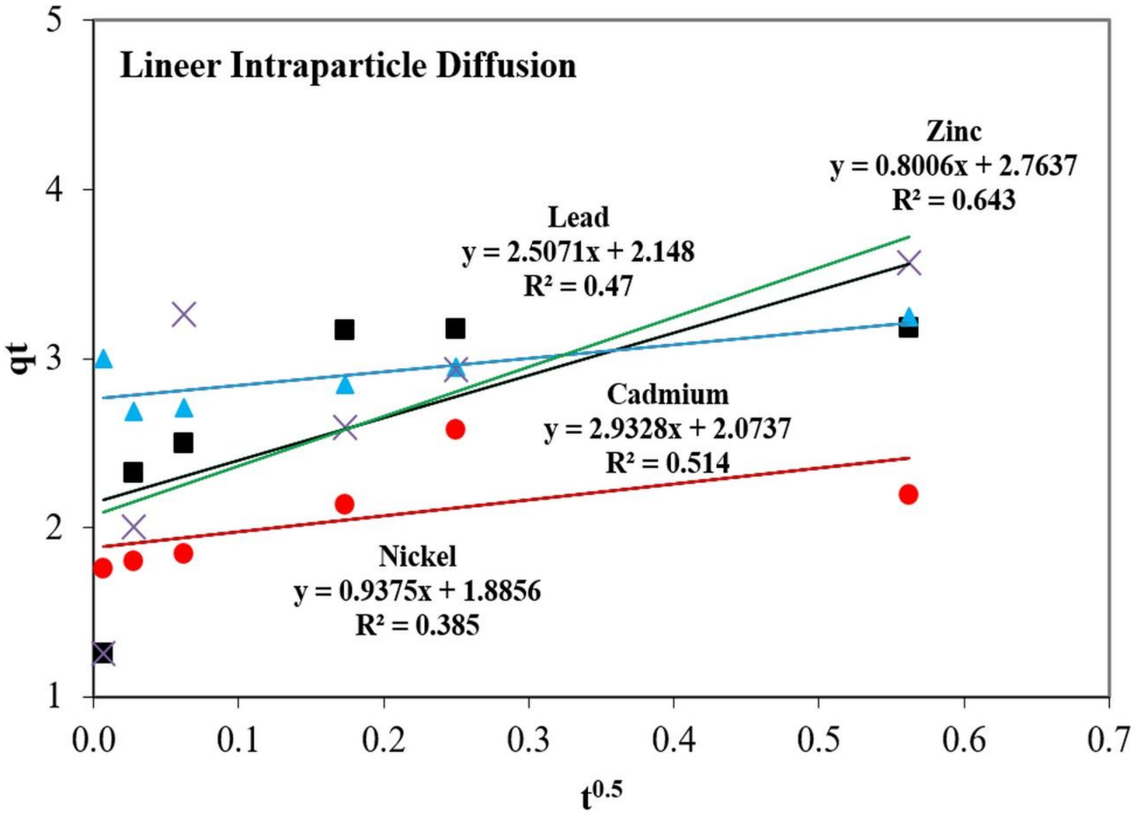

Sup. Figure 8. ID kinetic curves of Pb, Cd, Ni and Zn adsorption by BTW

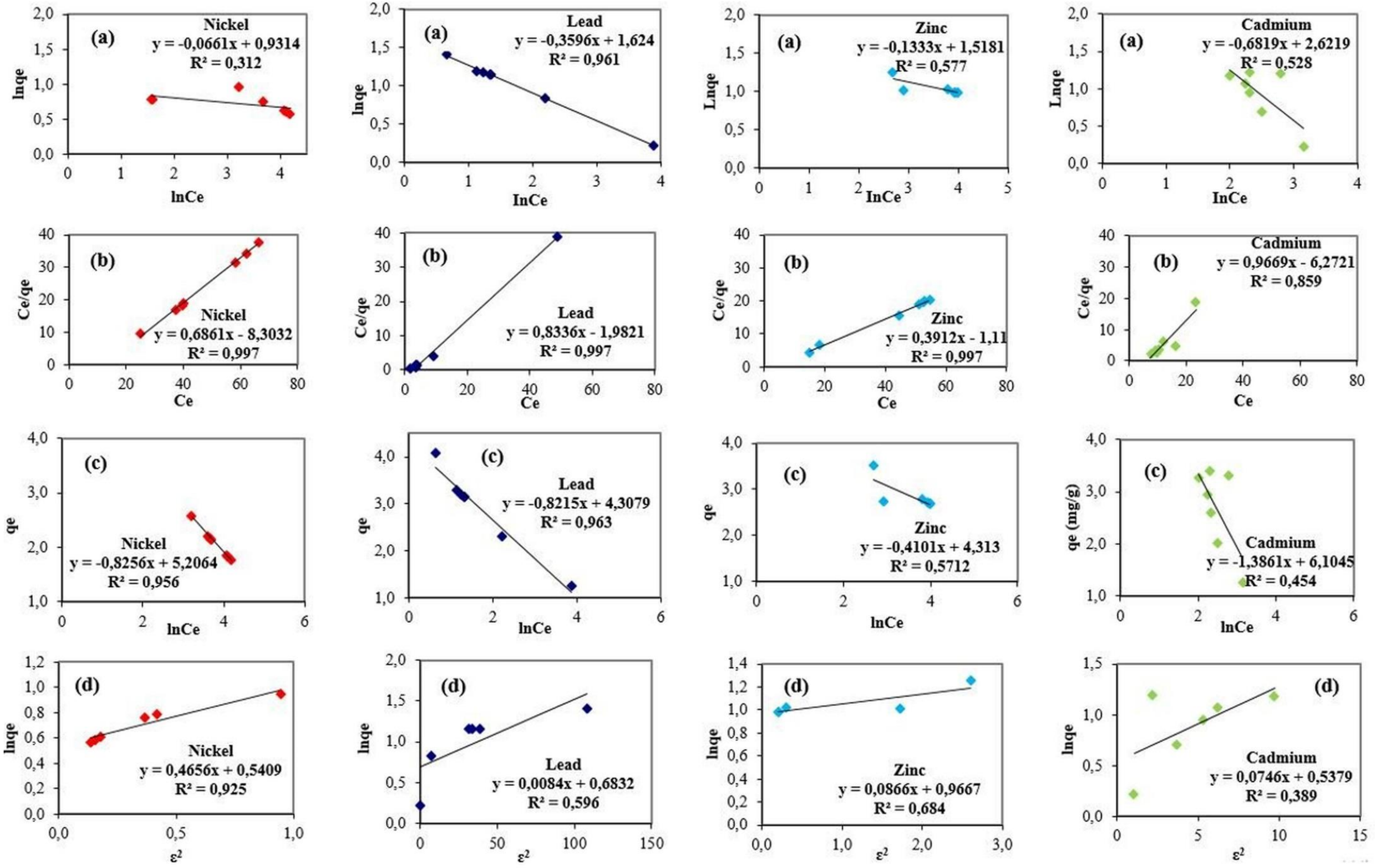

Sup. Figure 9. Isotherms curves of Pb, Cd, Ni and Zn adsorption by BTW **(a: Freundlich; b: Langmuir; c: Temkin; d: Dubinin-Radushkevich)**

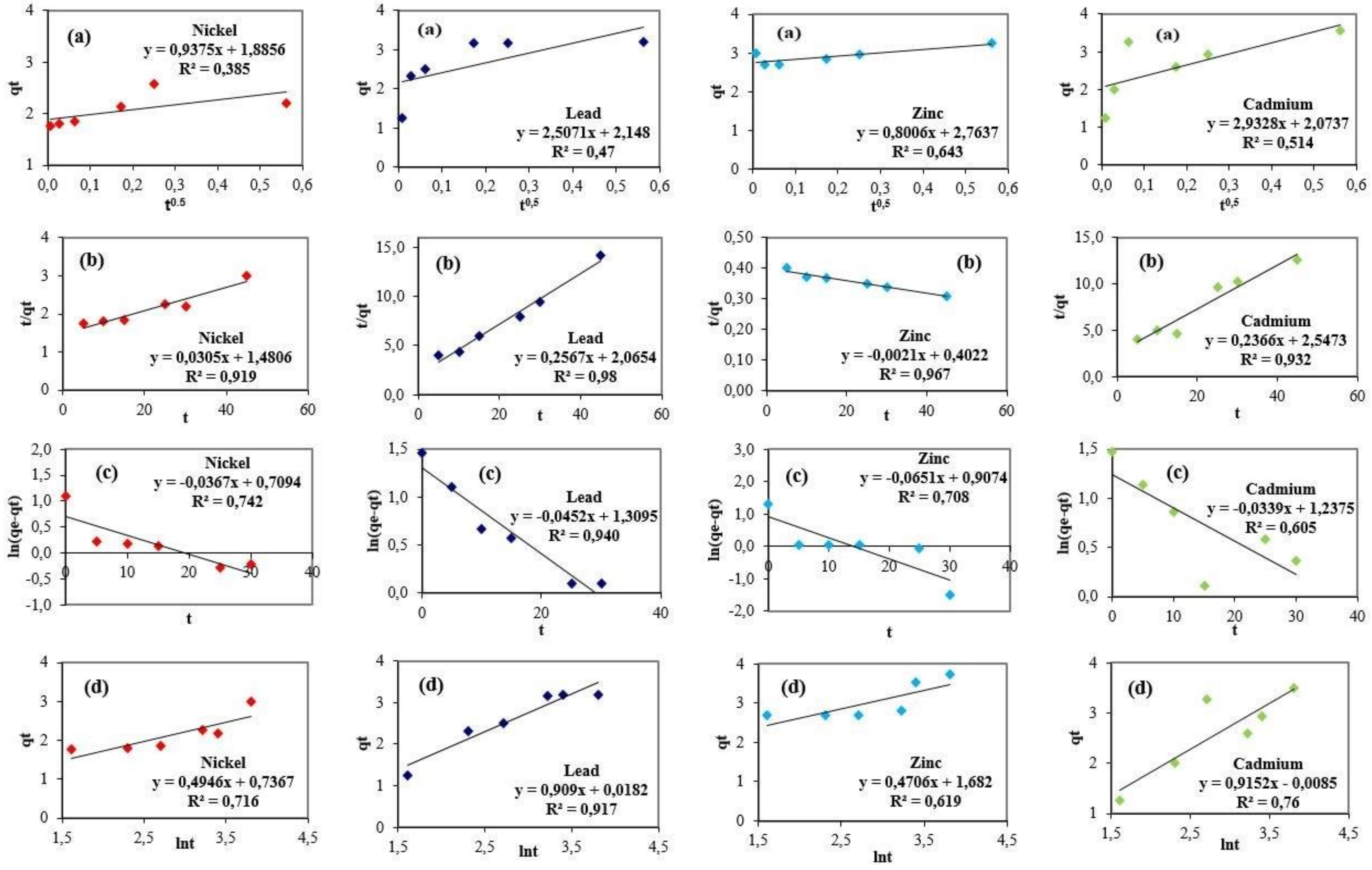

Sup. Figure 10. Kinetics curves of Pb, Cd, Ni and Zn adsorption by BTW **(a: Intraparticle diffusion; b: PSO; c: PFO; d: Elovich)**

pH values	% Removal			
	**Ni**	**Pb**	**Zn**	**Cd**
2	57,82±0,01^{e}	74,08±0,01^{e}	54,72±0,01^{e}	75,70±0,01^{e}
3	61,43±0,01^{d}	77,11±0,01^{d}	67,03±0,01^{d}	80,13±0,01^{a}
4	63,97±0,01^{c}	91,10±0,05^{a}	71,62±0,02^{c}	76,18±0,02^{b}
5	72,18±0,01^{a}	82,88±0,01^{b}	79,71±0,01^{a}	75,91±0,01^{d}
6	71,41±0,01^{b}	82,41±0,01^{c}	73,10±0,02^{b}	76,09±0,01^{c}

^{a,b,c,d,e}**Means ± SE**

Sup. Table 1. One-way ANOVA tests between pH values and % removals of Pb, Ni, Zn, Cd ions

Contact Time (min)	% Removal			
	**Ni**	**Pb**	**Zn**	**Cd**
1	1,53±0,01^{j}	77,13±0,01^{i}	17,37±0,01^{j}	23,50±0,01^{g}
2	12,78±0,01^{i}	97,97±0,01^{a}	24,98±0,01^{i}	49,61±0,01^{e}
5	17,84±0,01^{h}	89,10±0,07^{e}	27,94±0,01^{h}	82,18±0,01^{a}
10	55,44±0,01^{c}	89,43±0,01^{d}	84,74±0,01^{a}	56,14±0,01^{b}
30	76,34±0,01^{a}	89,82±0,01^{c}	71,17±0,01^{f}	47,97±0,01^{f}
45	63,56±0,01^{b}	90,03±0,01^{b}	70,44±0,01^{g}	49,71±0,01^{e}
60	54,71±0,01^{d}	89,12±0,07^{e}	80,20±0,01^{b}	49,80±0,01^{e}
90	50,15±0,01^{e}	88,40±0,01^{f}	76,92±0,01^{c}	51,24±0,01^{d}
120	48,02±0,01^{f}	88,06±0,01^{g}	76,13±0,01^{d}	52,33±0,01^{c}
150	47,26±0,01^{g}	87,59±0,00^{h}	73,65±0,00^{e}	52,80±0,01^{c}

^{a,b,c,d,e,f,g,h,i,j}**Means ± SE**

Sup. Table 2. One-way ANOVA tests between contact times and % removals of Pb, Ni, Zn, Cd ions

BTW dose (g)	% Removal			
	**Ni**	**Pb**	**Zn**	**Cd**
0, 1	36,31±0,00^{h}	49,71±0,01^{g}	46,61±0,01^{f}	41,20±0,00^{h}
0,5	40,44±0,01^{g}	90,69±0,01^{f}	46,08±0,01^{g}	79,44±0,01^{g}
1	43,94±0,01^{f}	98,03±0,01^{a}	44,81±0,01^{h}	93,15±0,00^{d}
1,5	61,46±0,01^{e}	96,01±0,00^{e}	55,16±0,01^{b}	88,52±0,00^{f}
2	76,17±0,01^{a}	96,02±0,00^{e}	85,05±0,01^{a}	91,29±0,00^{e}
2,5	64,02±0,01^{b}	96,07±0,00^{d}	51,11±0,01^{c}	93,33±0,01^{c}
3	62,37±0,01^{c}	96,38±0,01^{c}	48,20±0,01^{e}	93,70±0,00^{a}
5	61,56±0,01^{d}	96,89±0,00^{b}	48,63±0,01^{d}	93,52±0,00^{b}

^{a,b,c,d,e,f,g,h}**Means ± SE**

Sup. Table 3. One-way ANOVA tests between BTW doses and % removals of Pb, Ni, Zn, Cd ions
